# What Underpins the Trophic Networks of the Plankton in Shallow Oxbow Lakes?

**DOI:** 10.1007/s00248-016-0833-6

**Published:** 2016-08-20

**Authors:** J. Kosiba, E. Wilk-Woźniak, W. Krztoń, M. Strzesak, A. Pociecha, E. Walusiak, K. Pudaś, E. Szarek-Gwiazda

**Affiliations:** 1 Institute of Nature Conservation, Department of Freshwater Biology, Polish Academy of Sciences, al. A. Mickiewicza 33, 31-120 Krakow, Poland; 2 Central Laboratory, Municipal Water and Sewage Company, Lindego 9, 30-148 Krakow, Poland

**Keywords:** Trophic networks, Ciliates, Zooplankton, Phytoplankton, Oxbow lakes

## Abstract

The aim of this study was to determine the relationships in the microbial trophic network underpinning them about communities of plankton ciliates in shallow oxbow lakes of the Vistula River in southern Poland (Jeziorzany 1, Jeziorzany 2, Piekary, Tyniec). The plankton components (phytoplankton, ciliates, zooplankton) were grouped by dietary preference. The studied oxbows differed in physicochemical parameters and in phytoplankton. Cyanobacteria dominated in the total biomass of phytoplankton in the Tyniec oxbow, big green algae (>30 μm) in Piekary and Jeziorzany 1, and euglenoids in Jeziorzany 2 oxbow. The dominance pattern of ciliates and zooplankton were similar in all oxbows. Algivorous ciliates were the main dominant ciliates, and among zooplankton the dominant ones were herbivores that feed on small algae (<30 μm). The oxbows differed significantly in total phytoplankton biomass, cyanobacteria biomass, euglenoid biomass, small green algae (<30 μm) biomass, total biomass of zooplankton, biomass of zooplankton feeding on bacteria + algae, and biomass of zooplankton feeding on big algae (>30 μm). There was no significant differences in ciliate biomass between oxbows. In redundancy analyses, the variability at the trophic groups of plankton was described by explanatory variables in 42.3 %, and positive relationships were found: e.g., between omnivorous zooplankton biomass, the biomass of ciliates feeding on bacteria + algae, and NH_4_ level; between euglenoid biomass and dinoflagellate biomass; and between cyanobacteria biomass and bacterivorous ciliate biomass. Spearman correlation analysis revealed several relationships between different groups of plankton. In general, phytoplankton group shows more connection among themselves and with different zooplankton groups, e.g., phytoplankton biomass with herbivorous zooplankton biomass (−0.33); and cyanobacteria biomass with dinoflagellate biomass (0.65). Ciliates showed more connections among their trophic groups (e.g., algivorous ciliate biomass with omnivorous ciliate biomass, 0.78) and with zooplankton trophic groups (e.g., biomass of algivorous + bacterivorous ciliates with biomass of predator zooplankton, −0.36). Simple correlations analysis revealed the trophic food web network connectivity among plankton organisms, indicating the flow of organic matter from phytoplankton to zooplankton and from ciliates to zooplankton. Our study sheds light on the trophic relations among plankton ciliates, which are neglected in research but often form a large percentage of zooplankton biomass. In the studied oxbows, ciliate forms 6.7 % of total zooplankton biomass in Jeziorzany 1 and up to 44.5 % of it in the Piekary oxbow.

## Introduction

Microorganisms are basic components functioning in all water ecosystems playing role in maintenance of nutrient cycles. Our understanding of aquatic microbial ecology, particularly the interactions in those trophic networks, is still far from sufficient. To study them, network analyses employ quantitative food web models which describe the energy flow of an ecosystem and provides information about how the nature of the ecosystem has changed over time.

This type of research is especially needed for oxbow lakes, one of the most endangered landscape elements, which are disappearing due to river regulation, dam building and alteration of rivers and floodplains [1]. Oxbows are important habitats and refuges for microorganisms [2, 3]; they increase biodiversity and play an important role in maintaining gene pools [4].

**Fig. 1:**
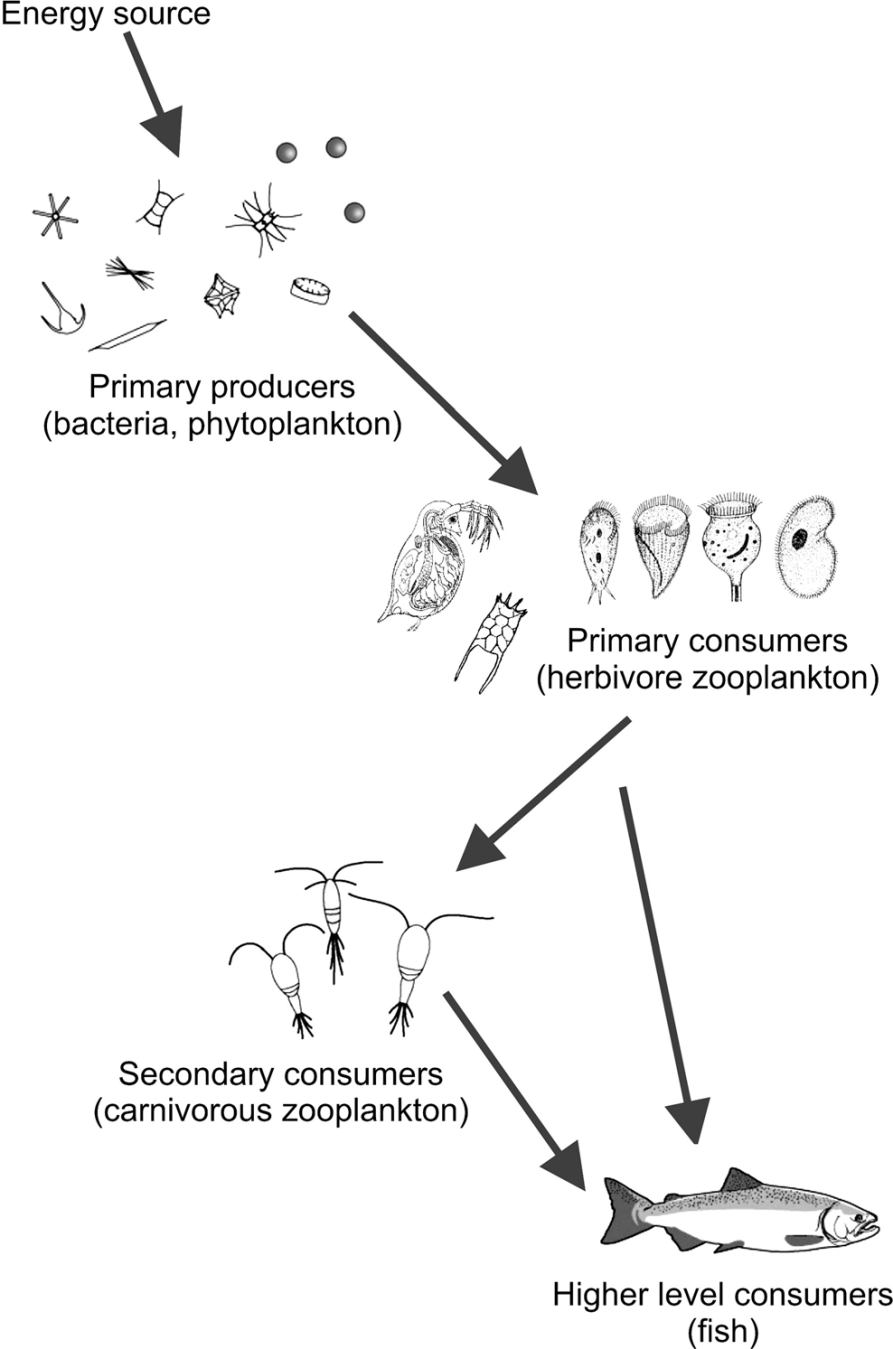
Scheme of trophic relationships in water ecosystems

Studies of the relationship among water organisms have a long history (e.g., [5–9]) and often focus on single relationship (in laboratory experiments; e.g., [10]) or simple trophic relationship (Fig. 1). For the management and maintenance of healthy water ecosystem, the interaction between the smallest components of trophic network in freshwaters must be known. Oxbow lakes tend to be naturally eutrophic. According to some authors, production in such ecosystems depends on “new nutrients,” and the classical pelagic food chain plays a more important role [11] than recycling of nutrients via microbial loops; the latter is more important in oligotrophic ecosystems [12], though some studies have confirmed the importance of microbial loops in eutrophic ecosystems as well [9, 13].

Thirty years ago, the PEG model [14] explained the role of abiotic and biotic factors as significant drivers of phytoplankton and zooplankton development in lakes, but today still we do not have a full grasp of the processes occurring in oxbow ecosystems. Because they are hydrologically variable, as lotic, lentic and semilotic types [15], the interactions among the components of their food webs are dependent on hydrological pulses [16]. A model of microbiological food web connections during different hydrological phases was recently proposed [17], but hydrological factors are not the only one regulating plankton relationships. Interbiotic relations between different components of plankton are also important.

The aim of this study was to determine the relationships in the trophic network of plankton components in shallow oxbow lakes, in order to improve our understanding of how carbon and energy is transferred among the microbial organisms inhabiting them.

## Materials and Methods

Samples were collected from four oxbow lakes of Poland’s largest river, the Vistula: Jeziorzany 1 (J1), Jeziorzany 2 (J2), Piekary (P) and Tyniec (T). These lakes are located in southern Poland in or near the city of Krakow, and are small, covering ca. 1.5–5.7 ha (Table 1).

**Table 1:** Geographical coordinates and chosen parameters of the studied oxbows

Parameter		Oxbows			
		J1	J2	P	T
Geographical coordinates		49°59′46.0″N 19°46′52.5″E	49°59′43.7″N 19°47′10.6″E	50°00′50.1″N 19°47′35.7″E	50°01′47.0″N 19°49′39.8″E
Area [ha]		2.21	2.19	1.56	5.75
Max. depth [m]		2.40	5.50	4.00	3.00
Temperature [°C]	Range (mean)	12.7–23.3 (18.5)	14.7–25.0 (20.7)	8.7–24.3 (17.3)	9.3–24.7 (17.9)
	CV	23	23	27	26
pH	Range	7.1–7.6	7.2–8.1	6.4–8.3	6.8–8.3
	CV	3	4	7	6
Oxygen saturation [%]	Range (mean)	27.4–94.6 (60.9)	75.7–115.2 (95.2)	53.1–100.8 (53.1)	41.0–169.6 (88.3)
	CV	40	14	24	43
Conductivity [μS cm^−1^]	Range (mean)	748–773 (802.0)	682–697 (690.8)	481–958 (653.0)	1268–1360 (1297.5)
	CV	1	1	19	2
HCO_3_^−^ [mg/L]	Range (mean)	229.8–306.9 (281.0)	202.9–280.2 (257.6)	196.4–265.1 (242.2)	224.9–317.0 (283.8)
	CV	11	12	8	11
SO_4_^2−^ [mg/L]	Range (Mean)	43.3–65.9 (52.9)	44.6–64.2 (51.8)	21.2–78.1 (36.7)	75.9–100.1 (84.7)
	CV	14	14	40	8
NO_3_^−^ [mg/L]	Range (mean)	0.23–0.95 (0.58)	nd-1.15 (0.47)	0.18–1.03 (0.39)	nd-1.06 (0.53)
	CV	53	110	62	46
NH_4_^+^ [mg/L]	Range (mean)	0.005–0.320 (0.140)	0.009–0.219 (0.071)	0.025–0.557 (0.183)	0.029–0.780 (0.220)
	CV	106	111	88	101
PO_4_^3−^ [mg/L]	Range (mean)	nd-0.030 (0.008)	nd-0.068 (0.026)	nd-0.190 (0.060)	nd-0.490 (0.150)
	CV	169	122	92	108
Mg^2+^ [mg/L]	Range (mean)	4.60–8.11 (7.04)	4.30–7.94 (6.91)	6.50–16.75 (13.06)	11.90–21.83 (18.94)
	CV	19	21	20	13
Chl *a* [μg/L]	Range (mean)	3.1–39.7 (21.2)	6.2–24.2 (13.2)	3.7–94.4 (32.3)	11.0–140.0 (37.3)
	CV	72	53	89	89

*n.d.* undetectable level, *CV* coefficient of variation

Samples were collected from the deepest part of each reservoir from May to October 2014, each month prior to cyanobacterial bloom formation and every week during bloom growth. We collected 108 samples for biological analyses (36 phytoplankton samples, 36 ciliate samples, 36 zooplankton samples). For physicochemical analyses, we collected 72 samples: 36 samples at 1 m depth and 36 samples near the lake bottom but finally used only the samples from 1 m depth for those tests. Water temperature, oxygen saturation, pH, conductivity and chlorophyll a concentration were measured in situ with a YSI 6600 V2 multiparameter sonde. Samples for analysis of anions (HCO_3_^−^, SO_4_^2−^, Cl^−^, NO_3_^−^, PO_4_^3−^) and cations (Ca^2+^, Mg^2+^, Na^+^, K^+^, NH_4_^+^) were immediately transported to the laboratory. Ion concentrations were measured with a Dionex Ion Chromatograph (DIONEX, IC25 Ion Chromatograph; ICS-1000, Sunnyvale, CA, USA) in the laboratory of the Institute of Nature Conservation, Polish Academy of Sciences.

Samples for biological parameters were taken from 1 m depth using a 5 L Ruttner sampler and were concentrated from 10 L with a plankton net (mesh size 10 μm for phytoplankton and ciliates, and 50 μm for the rest of zooplankton).

Since all the oxbows were relatively shallow and polymictic, no epilimnion, metalimnion, or hypolimnion were present. We took biological samples from 1 m depth because preliminary studies in previous years (unpubl. data) had shown that the diversity and biomass of plankton organisms, and especially phytoplankton, were highest at that depth, a finding supported by studies of ciliates and zooplankton: ciliates that are mixotrophic or consume algae prefer the upper levels of water [18, 19]; during the summer, the hypolimnetic refuge is not available to migratory zooplankton due to anoxic conditions [20].

Samples for quantitative analyses were immediately fixed with Lugol’s solution for algae and ciliates, and with 4 % formaldehyde for the rest of the zooplankton. Samples for phytoplankton, ciliates, and zooplankton (rotifers, cladocerans, copepods) were taken separately. Additional fresh samples, not fixed but concentrated as described above, were taken for species composition analysis of live material. Phytoplankton species were identified and counted in a modified chamber (0.4 mm high, 22 mm diameter). Phytoplankton biomass was calculated from the cell numbers and specific volumes [21].

Ciliates were determined taxonomically from living material in a 1-mL chamber with a glass cover, according to Foissner and Berger [22, 23]. The total biomass of ciliates (mg/L) was calculated according to Jerome et al. [24], Menden-Deuer and Lessard [25], Wiąckowski et al. [26] and Putt and Stoecker [27].

Zooplankton samples were analyzed in a 0.5-mL chamber. Average of five counts were calculated. The species were identified with keys [28–31]. Dry weight was calculated using a regression equation for body length and weight for each species [32–36]. Because phytoplankton and ciliates were calculated as fresh biomass, zooplankton dry mass was recalculated according to the index proposed by Bottrell et al. [34].

The above analyses employed a Nikon H550L light microscope at 40–1000 × .

To describe the network structure, microorganisms were divided by trophic group: primary producers (phytoplankton), protozoan consumers (ciliates), and metazoan consumers (zooplankton - rotifers, cladocerans, copepods). Producers were subdivided into size and trophic classes: cyanobacteria (only large colonies or trichomes were present in the collected samples), big diatoms (>30 μm), small diatoms (<30 μm), big green algae (>30 μm), small green algae (<30 μm), and mixotrophic algae. Mixotrophic algae were grouped as follows: cryptophytes (sparse phagotrophic species), golden brown algae (equal use of phagotrophy and phototrophy; e.g., *Dinobryon* [37]), dinophytes, and euglenoids. Ciliates were grouped as follows: species that feed on algae, bacterivorous species, algivorous and bacterivorous species, and omnivorous species [22, 23]. Zooplankton group was divided into species that feed on the seston and bacteria, species that feed on algae >30 μm, species that feed on algae <30 μm, predators, and omnivorous species [38].

The basic statistics used for data analysis were range (minimum–maximum), average, standard deviation (SD) and coefficient of variation (CV). The Kruskal–Wallis test was used to determine the significance of differences in biomass between the different plankton components of oxbows. Spearman correlations were used to build a model to explain the relationships between plankton components, and redundancy analysis (RDA) was used to build a model to explain the relationships between plankton components and physicochemical parameters. Statistica 10.0 and CANOCO 5 for Windows were used for these statistical analyses. The data were log-transformed. The manual forward selection procedure was run using the Monte Carlo permutation test. Variables having a conditional effect that was significant at *p* < 0.05 were included.

## Results

### Physicochemical Factors

All the oxbows are in the same geographical zone and are exposed to the same climate, but showed differences in physicochemical parameters (Table 1).

The shallowest oxbow was J1 (2.4 m) and the deepest was J2 (5.5 m). Table 1 represents the parameters bearing any relation to plankton components as assessed by RDA. Variation (CV) of water temperature in J1 and J2 was similar, and was higher in P oxbow and T oxbow. Water pH showed a similar tendency. Variation of oxygen saturation was highest in J1 and T. Mean conductivity was highest in the water of T, and variation of conductivity was highest for P. Mean NH_4_^+^ and PO_4_^3−^ concentrations were highest in T, and NO_3_^−^ was highest in J1. Other parameters also differed oxbows from each other.

### Phytoplankton

The phytoplankton consisted of cyanobacteria, golden brown algae, cryptomonads, dinoflagellates, euglenoids, diatoms, and green algae. Golden brown algae and cryptomonads were found only occasionally in single samples. The mean total biomass of phytoplankton was highest in T and lowest in J2. Variation of total phytoplankton biomass was highest in P (Table 2).

**Table 2:** Biomass (mg/L) of phytoplankton, plankton ciliates and zooplankton in oxbows—basic statistics

	J1			J2			P			T		
Statistic	Phyto	Ciliates	Zoo	Phyto	Ciliates	Zoo	Phyto	Ciliates	Zoo	Phyto	Ciliates	Zoo
Min-max	4.8–28.5	0.07–1.1	1.9–9.3	1.8–12.4	0.1–2.6	0.5–6.3	1.0–30.6	0.1–26.7	4.1–19.1	3.9–163.3	0.07–12.0	4.0–12.4
Average	14.8	0.5	6.7	6.5	1.0	4.0	11.9	8.0	9.9	65	3.7	8.1
SD	9.9	0.4	2.6	4.4	0.9	1.9	10.8	9.5	4.5	44. 5	3.9	2.8
CV (%)	67	75	39	68	90	48	90	119	46	69	108	34

*SD* standard deviation, *CV* coefficient of variation

**Fig. 2:**
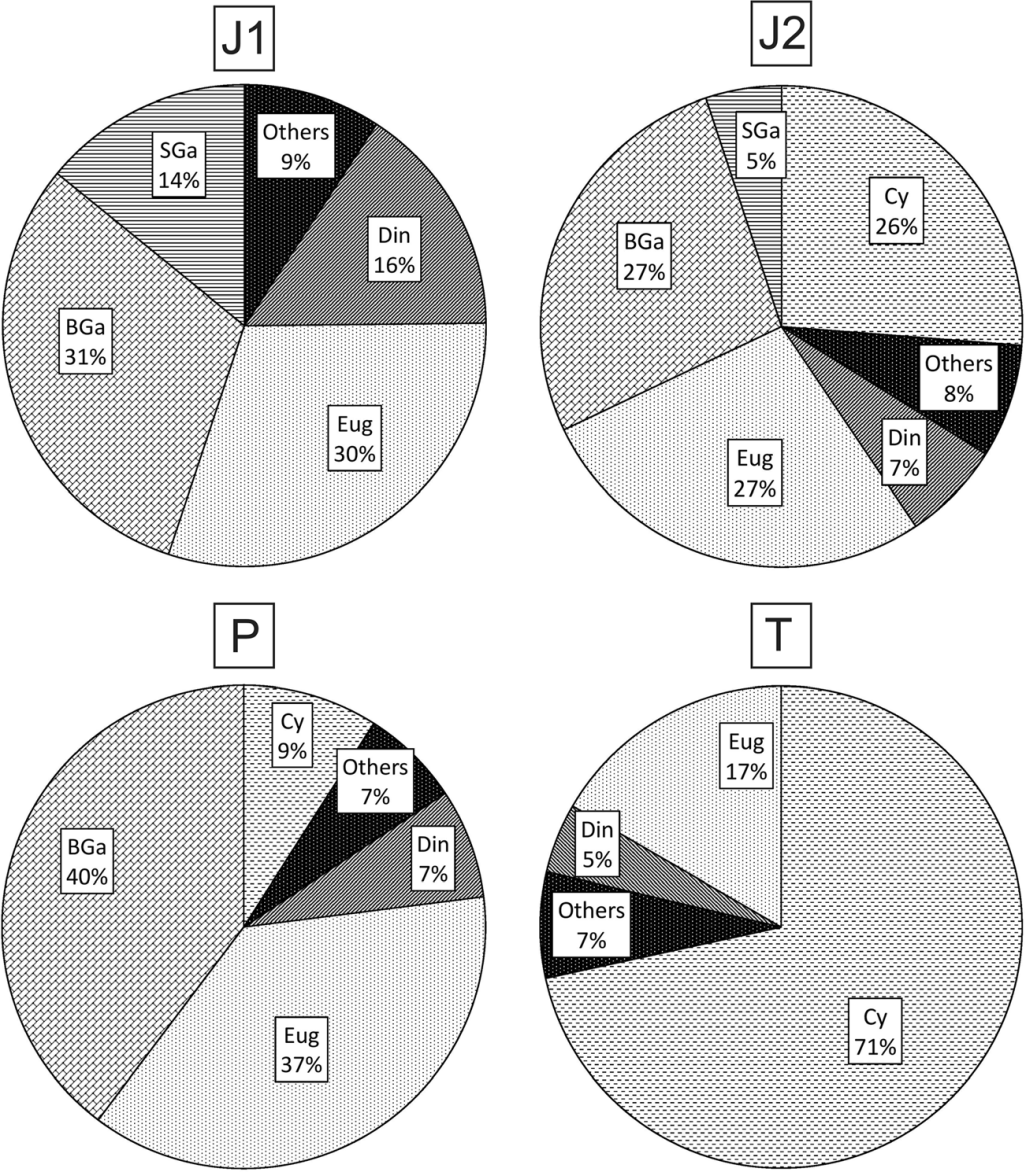
Percentage shares of different phytoplankton groups in total phytoplankton biomass in the four studied oxbow lakes. Abbreviations: *J1: Din* dinoflagellates, *Eug* euglenoids, *BGa* big green algae, *SGa* small green algae, *Others* cyanobacteria, golden brown algae, diatoms. *J2: Din* dinoflagellates, *Eug* euglenoids, *BGa* big green algae, *SGa* small green algae, *Cy* cyanobacteria, *Others* golden brown algae, diatoms. *P: Din* dinoflagellates, *Eug* euglenoids, *BGa* big green algae, *Cy* cyanobacteria, *Others* small green algae, golden brown algae, diatoms, cryptomonads. *T: Cy* cyanobacteria, *Din* dinoflagellates, *Eug* euglenoids, *Others* green algae, diatoms, cryptomonads

The pattern of dominance in the total biomass of phytoplankton was somewhat similar for J1 and J2, however differed between the oxbows (Fig. 2):

- J1: big green algae > euglenoids > dinoflagellates > small green algae.
- J2: euglenoids = big green algae > cyanobacteria.
- P: big green algae > euglenoids > cyanobacteria > dinoflagellates.
- T: cyanobacteria > euglenoids > dinoflagellates.

### Ciliates

The plankton ciliates consisted of the following groups: (1) algivorous ciliates (*Oligotrichida*: *Codonella cratera*, *Tintinidium* sp.; *Prostomatida*: *Coleps spetai*); (2) bacterivorous ciliates (*Peritrichia*: *Epistylis* sp., *Vorticella* sp.; *Hypotrichia*: *Aspidisca* sp.); (3) mixed type of feeding – ciliates that feed on algae and bacteria (*Oligotrichida*: *Strobilidium* sp.; *Peritrichia*: *Vorticella campanula*); and (4) omnivorous species (*Hymenostomata*: *Cinetochilum margaritaceum*, *Paramecium bursaria*; *Hypotrichia*: *Euplotes patella*; *Prostomatida*: *Coleps hirtus*: *Heterotrichida*: *Stentor* sp.). Mean total biomass of plankton ciliates and variation of total biomass were highest for P and lowest for J1 (Table 2).

**Fig. 3:**
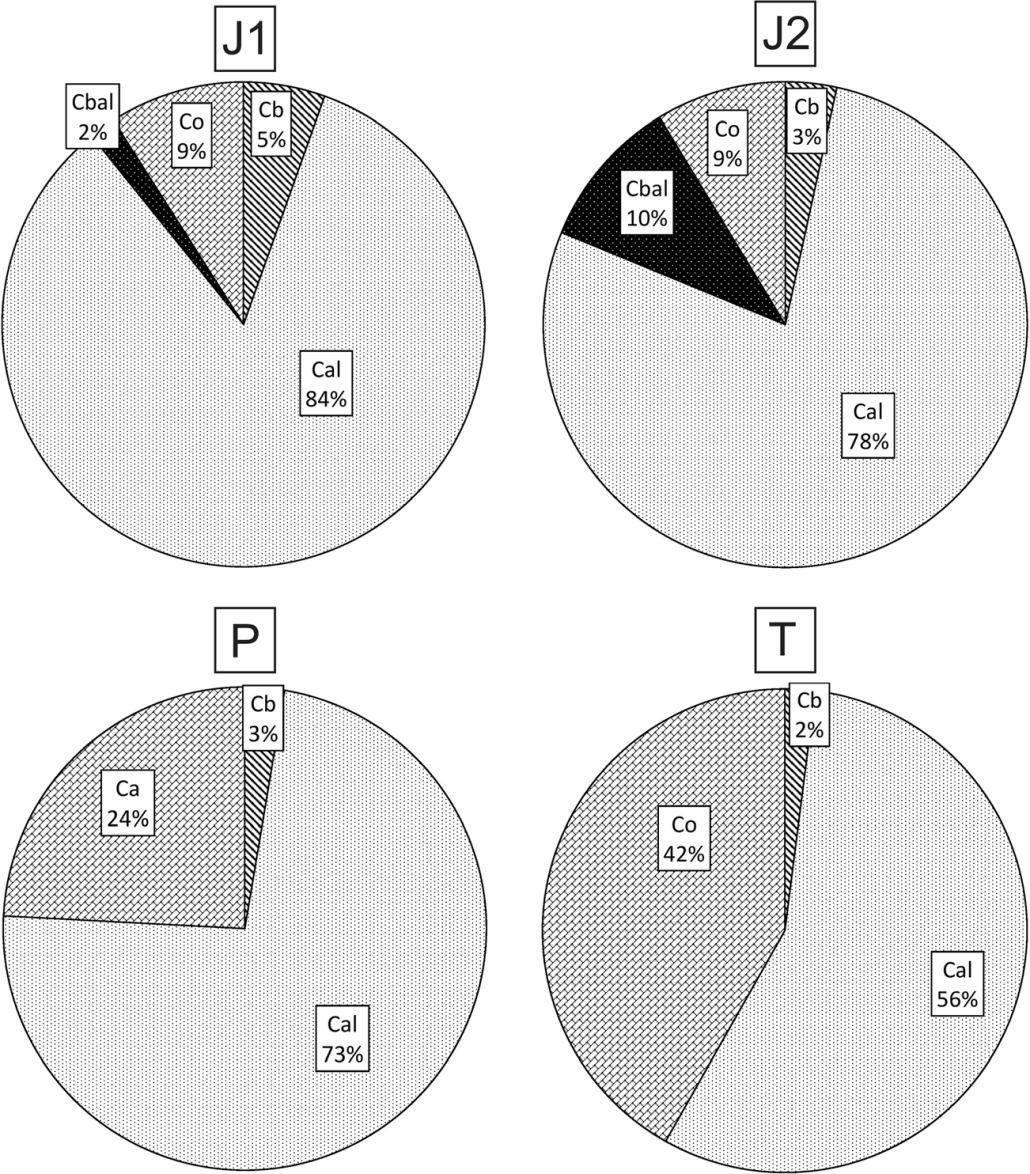
Percentage shares of different ciliate groups in total ciliate biomass in the four studied oxbow lakes. Abbreviations: *J1: Cal* algivorous ciliates, *Cbal* algivorous and bacterivorous ciliates, *Co* omnivorous ciliates, *Cb* bacterivorous ciliates, *J2: Cal* algivorous ciliates, *Cbal* algivorous and bacterivorous ciliates, *Co* omnivorous ciliates, *Cb* bacterivorous ciliates, *P: Cal* algivorous ciliates, *Co* omnivorous ciliates, *Cb* bacterivorous ciliates. *T: Cal* algivorous ciliates, *Co* omnivorous ciliates, *Cb* bacterivorous ciliates

The pattern of dominance in the total biomass of ciliates was similar for all oxbows (Fig. 3):

- J1: algivorous ciliates > omnivorous ciliates > bacterivorous ciliates > algivorous and bacterivorous ciliates
- J2: algivorous ciliates > algivorous and bacterivorous ciliates > omnivorous ciliates > bacterivorous ciliates
- P: algivorous ciliates > omnivorous ciliates > bacterivorous ciliates
- T: algivorous ciliates > omnivorous ciliates > bacterivorous ciliates

For all oxbows taken together, algivorous ciliates were dominant, followed by omnivorous ciliates. Bacterivorous and bacterio-algivorous ciliates had lower shares of total ciliate biomass.

### Zooplankton

Zooplankton consisted of the following trophic groups: (1) seston-feeding and bacterivorous animals (rotifers: *Brachionus angularis*, *B. diversicornis*, *B. urceolaris*, *Filinia longiseta*, *Keratella cochlaris*, *K. tecta*, *Polyarthra major*, *P. remata*, *P. vulgaris*, *Pompholyx sulcata*; copepods: nauplii), 2) herbivorous animals that feed on small algae (<30 μm) (rotifers: *Brachionus calyciflorus*, *Kellicotia longispina*, *Keratella quadrata*, *Trichocerca similis*; cladocerans: *Bosmina longirostris*, *Chydorus sphaericus*, *Diaphanosoma brachyurum*, *Eubosmina coregoni*, *E. gibera*, *E. longispina*, *Moina micrura*; copepods: *Acanthocyclops venustus*, *Cyclops vicinus*, *Eurytemora affinis*, copepodites); (3) herbivorous animals that feed on algae larger than 30 μm (cladocerans: *Daphnia ambigua*, *D. cucullata*, *D. longispina*, copepods: *Eudiaptomus gracilis*); (4) predators (cladocerans: *Leptodora kindtii*; copepods: *Cyclops abyssorum*, *C. strennus*, *Thermocyclops crassus*); and (5) omnivorous species (rotifers: *Asplanchna priodonta*, *Gastropus minor*, *Trichocerca capucina*; copepods: *Mesocyclops leuckartii*, *Metacyclops gracilis*).

Variation of total zooplankton biomass was highest for J2 and P, and lowest for T (Table 2).

**Fig. 4:**
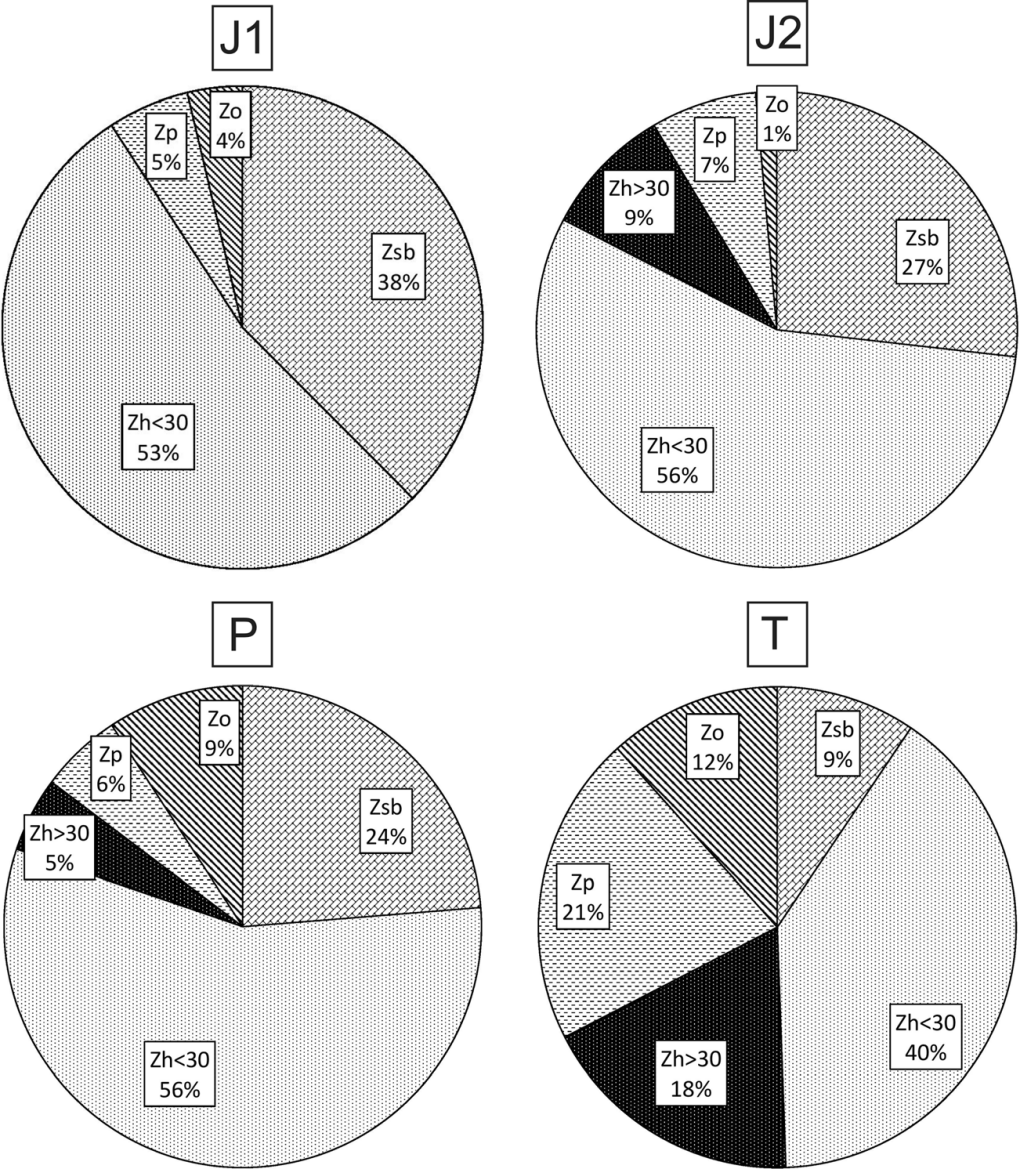
Percentage shares of different zooplankton groups in total zooplankton biomass in the four studied oxbow lakes. Abbreviations: *J1: Zsb* seston and bacterivorous animals, *Zh < 30* herbivorous animals that feed on small algae, *Zp* predators, *Zo* omnivorous zooplankton. *J2: Zsb* seston and bacterivorous animals, *Zh < 30* herbivorous animals that feed on small algae, *Zh > 30* herbivorous animals that feed on big algae, *Zp* predators, *Zo* omnivorous zooplankton. *P: Zsb* seston and bacterivorous animals, *Zh < 30* herbivorous animals that feed on small algae, *Zh > 30* herbivorous animals that feed on big algae, *Zp* predators, *Zo* omnivorous zooplankton. *T: Zsb* seston and bacterivorous animals, *Zh < 30* herbivorous animals that feed on small algae, *Zh > 30* herbivorous animals that feed on big algae, *Zp* predators, *Zo* omnivorous zooplankto

Herbivores that feed on algae smaller than 30 μm were dominant in all oxbows. Three oxbows (J1, J2, P) showed a similar pattern of dominant species; T differed from the others (Fig. 4):

- J1: herbivores that feed on small algae (<30 μm) > seston-feeding and bacterivorous animals > predators > omnivores.
- J2: herbivorous animals that feed on small algae > seston-feeding and bacterivorous animals > herbivorous animals that feed on big algae > predators > omnivores.
- P: herbivorous animals that feed on small algae > seston-feeding and bacterivorous animals > omnivores > predators > herbivorous animals that feed on big algae.
- T: herbivorous animals that feed on small algae > predators > herbivorous animals that feed on big algae > omnivores > seston-feeding and bacterivorous animals.

### Total Plankton

There were significant differences in total phytoplankton biomass between J2 and T and between P and T (Table 3), in cyanobacterial biomass between J1 and T, in euglenoid biomass between J2 and T, in the biomass of small green algae between J1 and P and between P and T, in total zooplankton biomass between J2 and T, in the biomass of zooplankton that feeds on the seston and bacteria between J1 and T and between P and T, and in the biomass of zooplankton that feeds on big algae between J1 and T. Neither total ciliate biomass nor the biomass of any ciliate group differed between oxbows.

**Table 3:** Statistically significant differences between various components of plankton and between oxbows (Kruskal–Wallis test; *z* statistic value; *p* level of significance)

Biomass	Oxbow lake	*z*	*p*
Total biomass of phytoplankton H (3, *N* = 36) = 13.56	Jeziorzany 2 - Tyniec	3.097	0.012
	Piekary - Tyniec	2.979	0.017
Biomass of cyanobacteria H (3, *N* = 36) = 13.14	Jeziorzany 1- Tyniec	3.336	0.005
Biomass of euglenoids H (3, *N* = 36) = 8.77	Jeziorzany2 - Tyniec	2.721	0.039
Biomass of small green algae H (3, *N* = 36) = 22.65	Jeziorzany 1 - Piekary	3.970	<0.000
Total biomass of zooplankton H (3, *N* = 36) = 11.44	Jeziorzany 2 - Piekary	3.315	0.006
Biomass of zooplankton feed on bacteria + algae H (3, *N* = 36) = 11.95	Jeziorzany1 - Tyniec	2.707	0.041
	Piekary - Tyniec	2.830	0.028
Biomass of zooplankton feed on big algae H (3, *N* = 36) = 14.96	Jeziorzany 1 - Tyniec	3.831	<0.000

### Statistical Analysis

**Fig. 5:**
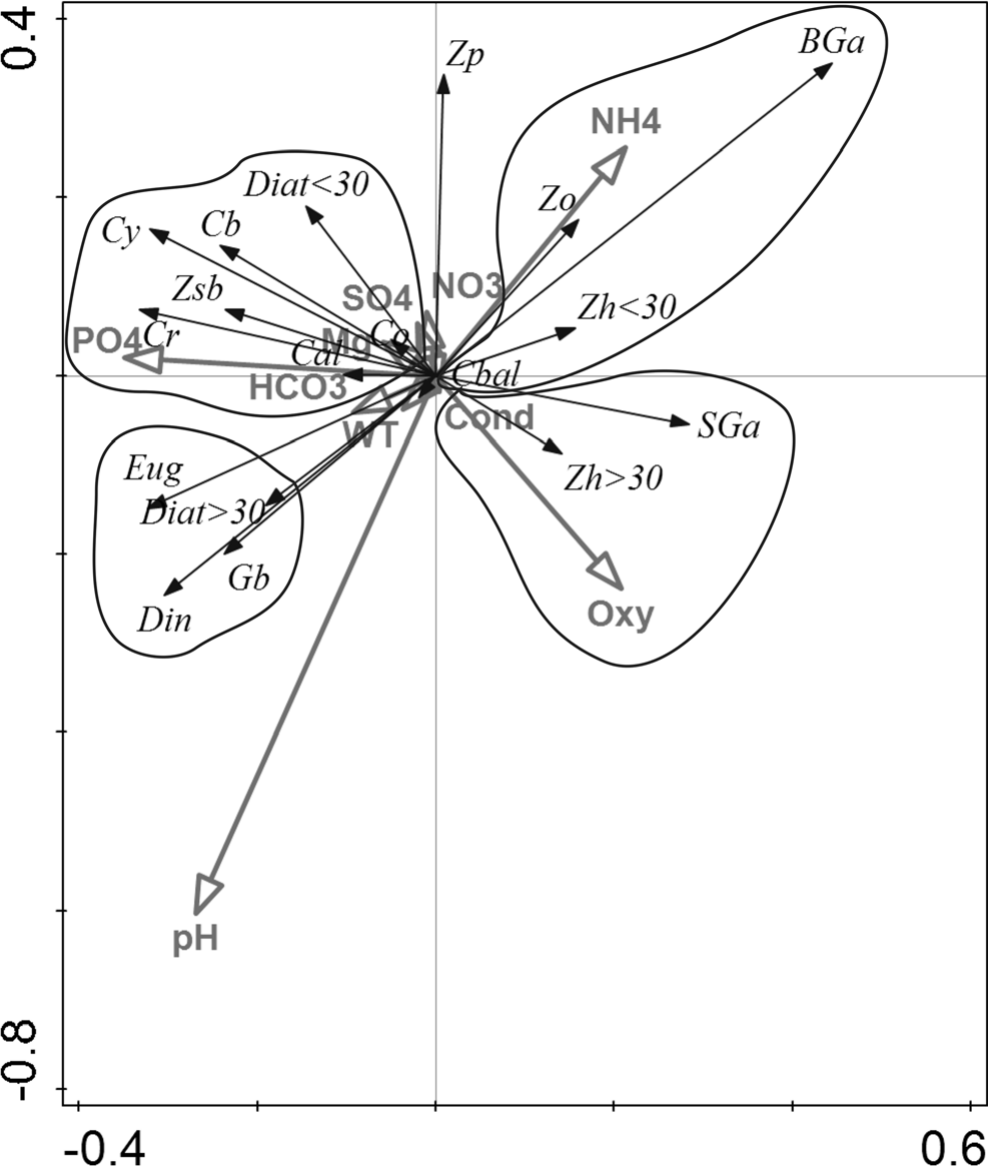
Redundancy analysis (RDA) biplot of the relationships between trophic groups of plankton components and environmental factors (constrained partial analysis, partial RDA). Partial variation was 110.9344; the explanatory variables accounted for 42.3 %; adjusted explained variation was 1.1 %; eigenvalues: 0.0765 (axis 1); 0.0311 (axis 2), 0.0229 (axis 3), 0.0194 (axis 4); explained variation (cumulative): 17.54; 24.67; 29.92; 34.36; pseudocanonical correlations: 0.8543; 0.7360; 0.7225; 0.5692; explained fitted variation (cumulative): 41.48; 58.33; 70.74; 81.24; permutation test results: on first axis, pseudo-*F* = 3.0, *P* = 0.186; on all axes, pseudo-*F* = 1.0, *P* = 0.440. Abbreviations: *Din* dinoflagellates, *Eug* euglenoids, *BGa* big green algae, *SGa* small green algae, *Cy* cyanobacteria, *Gb* golden brown algae, *Diat > 30* big diatoms, *Diat < 30* small diatoms, *Cr* cryptomonads. *Cal* algivorous ciliates, *Cbal* algivorous and bacterivorous ciliates, *Co* omnivorous ciliates, *Cb* bacterivorous ciliates. *Zp* predator zooplankton, *Zo* omnivorous zooplankton, *Zh < 30* herbivorous zooplankton that feeds on small algae, *Zh > 30* herbivorous zooplankton that feeds on big algae, *Zsb* zooplankton that feeds on the seston and bacteria

Spearman correlation revealed several relationships between different groups of plankton (Table 4). RDA analysis showed relationship between different groups and abiotic parameters. The explanatory variables described 42.3 % variability at plankton trophic groups in oxbow lakes (Fig. 5). We noted the following groups of positive relationship: (*a*) the biomass of big green algae, the biomass of herbivorous zooplankton that feeds on small algae (<30 μm), the biomass of omnivorous zooplankton, the biomass of ciliates that feed on bacteria and algae, and the concentration of NH_4_^+^; (*b*) the biomass of small green algae, the biomass of zooplankton that feeds on big algae (>30 μm), conductivity, and oxygen concentration; (*c*) the biomass of euglenoids, the biomass of big diatoms, the biomass of dinoflagellates, and the biomass of golden brown algae; (*d*) the biomass of cyanobacteria and cryptomonads, the biomass of small diatoms, the biomass of bacterivorous ciliates, the biomass of algivorous ciliates, the biomass of omnivorous ciliates, the biomass of zooplankton that feeds on the seston and bacteria, and the concentrations of PO_4_^3−^, SO_4_^2−^, HCO_3_^−^, and Mg^2+^; (*e*) the biomass of predator zooplankton was correlated with the NO_3_^−^ concentration. Negative relationships were found between groups *a* and *c* and between groups *b* and *d*.

**Table 4:** Statistically significant Spearman correlations between various trophic groups of plankton occurring in the studied oxbow lakes (*p* < 0.05)

Biomass	Biomass	Coefficient
Phytoplankton in total	Herbivorous animals feed on small algae (dimension < 30 μm)	−0.33
	Herbivorous animals feed on big algae (dimension > 30 μm)	0.36
	Predator zooplankton	0.49
Ciliates in total	Euglenoids	0.33
Zooplankton in total	Golden brown algae	−0.33
	Algae- and bacterivorous ciliates	−0.63
Algivorous ciliates	Omnivorous ciliates	0.78
Algae- and bacterivorous ciliates	Zooplankton in total	−0.63
	Predator zooplankton	−0.36
	Herbivorous animals feed on small algae	−0.47
Omnivorous ciliates	Algivorous ciliates	0.78
	Herbivorous animals feed on small algae	0.45
	Cryptomonads	0.35
	Euglenoids	0.41
Zooplankton feed on seston + bacteria	Herbivorous animals feed on big algae	−0.33
Herbivorous animals feed on algae smaller dimension than 30 μm	Phytoplankton in total	−0.34
	Algae- and bacterivorous ciliates	−0.47
	Omnivorous ciliates	0.45
	Cyanobacteria	−0.33
	Dinoflagellates	−0.37
	Small green algae (dimension < 30 μm)	−0.45
Herbivorous animals feed on algae bigger dimension than 30 μm	Phytoplankton in total	0.36
	Zooplankton feed on seston + bacteria	−0.34
	Predator zooplankton	0.42
	Cyanobacteria	0.43
	Euglenoids	0.40
Predator zooplankton	Phytoplankton in total	0.49
	Algae- and bacterivorous ciliates	−0.36
	Herbivorous animals feed on big algae	0.42
	Cyanobacteria	0.37
	Golden brown algae	−0.37
	Big green algae (dimension >30 μm)	0.51
Cyanobacteria	Golden brown algae	−0.35
	Dinoflagellates	0.65
Dinoflagellates	Euglenoids	0.47

## Discussion

In the cascade model, the structure of the food web is determined by the trophic position of the component species: species in a higher trophic position can consume only species that occupy a lower position. The theoretical cascade model has been adopted in empirical studies, and now the trophic positions of species are commonly used to estimate food web structure and trophic connectivity [39]. Based on the biomass of various components of the plankton and the biomass of trophic groups, we constructed a model of the trophic network in small, shallow oxbow lakes.

Phytoplankton forms the first trophic level that directly responds to changes in abiotic parameters [40]. Differences in physicochemical parameters such as conductivity and the concentrations of phosphates, nitrate nitrogen, and ammonia nitrogen resulted in clear differences in phytoplankton composition between the studied oxbow lakes. The differences at higher levels (e.g., ciliates, zooplankton) were not as conspicuous. We found differences in ciliate biomass and variability, but the dominance of trophic groups of ciliates was similar in the different oxbows, as the dominance of zooplankton trophic groups, which differed only for the zooplankton in the Tyniec oxbow (highest trophic status). There were significant differences in the biomass of trophic groups between the oxbows for some phytoplankton and zooplankton groups, but not for ciliates. It appears that ciliates are generalists, in that they can consume multiple resources [41]. Pelagic ciliates are the main component of the microzooplankton, forming up to 34 % of the total zooplankton biomass in eutrophic lakes and up to 62 % of it in hypertrophic lakes [42, 43]. In our study, the share of plankton ciliates in total zooplankton biomass ranged from 6.7 % in Jeziorzany 1 to 44.5 % in the Piekary oxbow.

**Fig. 6:**
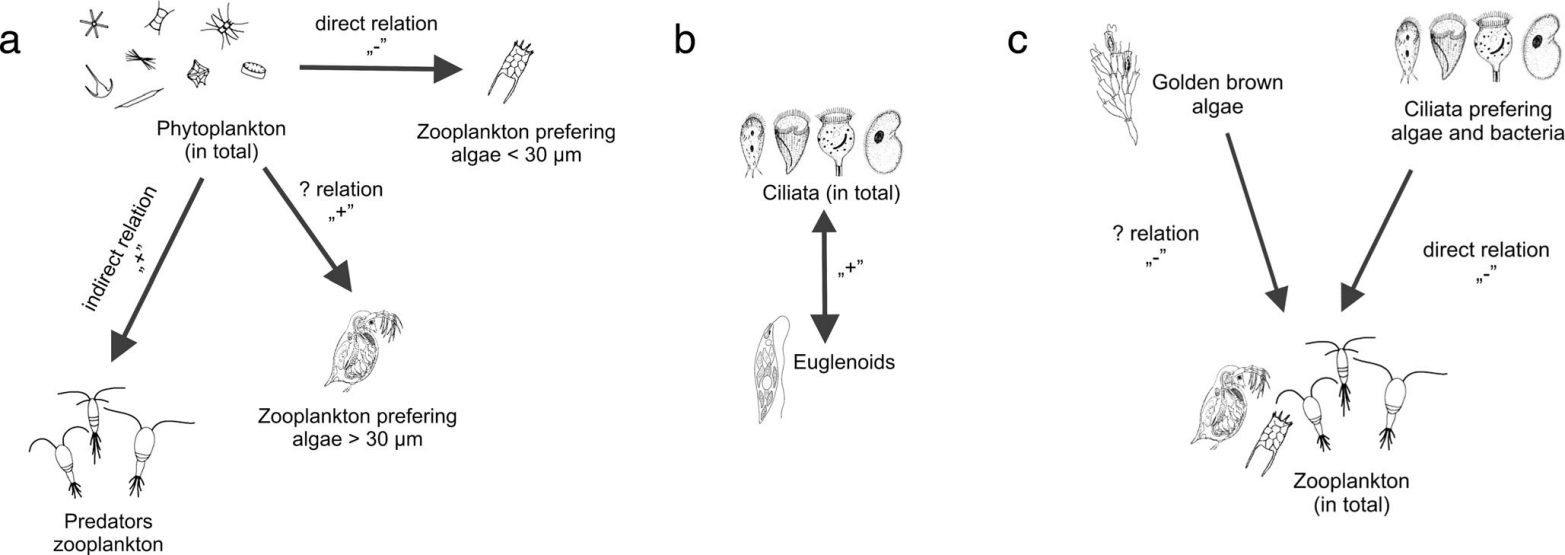
Model of the relationships between the plankton in oxbow lakes (only significant ones shown): **a** total biomass of phytoplankton; **b** total biomass of ciliates; and **c** total biomass of zooplankton

**Fig. 7:**
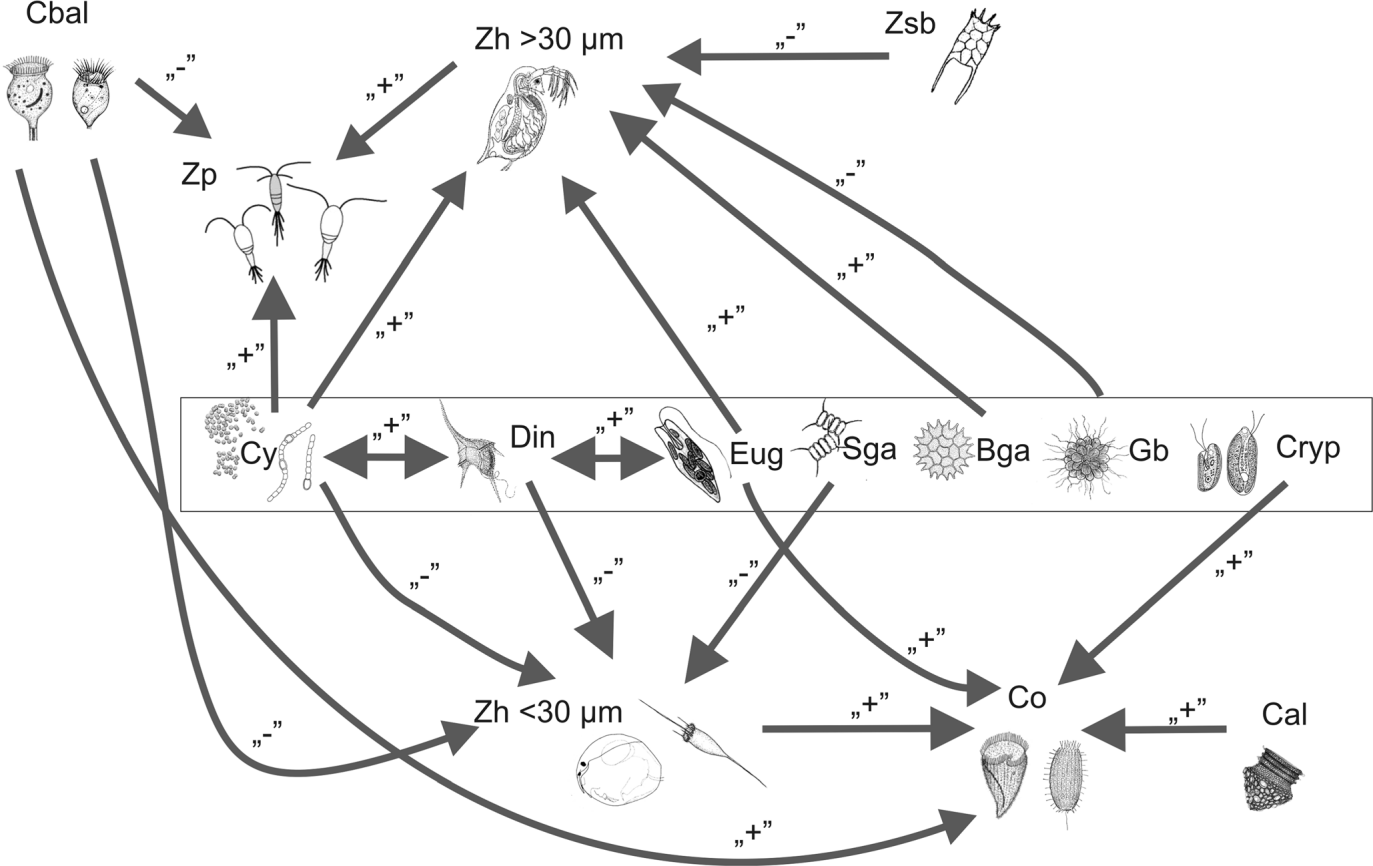
Model of the trophic network among plankton components in small shallow oxbow lakes. Abbreviations: *Din* dinoflagellates, *Eug* euglenoids, *BGa* big green algae, *SGa* small green, *Cy* cyanobacteria, *Gb* golden brown algae, *Cr* cryptomonads. *Cal* algivorous ciliates, *Cbal* algivorous and bacterivorous ciliates, *Co* omnivorous ciliates. *Zp* predator zooplankton, *Zh < 30 μm* herbivorous zooplankton that feeds on small algae, *Zh > 30 μm* herbivorous zooplankton that feeds on big algae, *Zsb* zooplankton that feeds on the seston and bacteria; "-" negative relation, "+" positive relation

In redundancy analysis, physicochemical factors explained 42.3 % of the variability in the trophic groups of plankton. Simple correlations allowed us to delineate trophic network connectivity among the plankton organisms, implying direct and indirect relationships such as competition, predation, coexistence, disturbance, and resource heterogeneity (Fig. 6a–c and 7). Predation was shown by a negative correlation between total phytoplankton biomass and the biomass of herbivorous zooplankton that feeds on small algae (<30 μm). An indirect relationship was seen between total phytoplankton biomass and the biomass of predator zooplankton; the positive correlation suggests an undisclosed link (herbivorous animals) between phytoplankton and predators. The positive correlation between total phytoplankton biomass and the biomass of zooplankton that feeds on big algae (>30 μm) indicates that an increase in zooplankton that feeds on big algae promotes an increase in the total biomass of phytoplankton, and vice versa.

The positive correlation between the total biomass of ciliates and that of euglenoids (Figs. 6b and 7) is explained by their coexistence or by their food resource heterogeneity (heterotrophy and autotrophy). Moreover, both groups are mobile and can seek food by moving in the water.

The negative correlation between total zooplankton biomass and the biomass of ciliates that feed on algae + bacteria (Figs. 6c and 7) showed a direct relationship reflecting predation of plankton animals on ciliates. Field and laboratory experiments have shown that the impact of grazing on the ciliate stock by copepods is greatest when the phytoplankton concentration is low and when it is dominated by small phytoflagellates [44].

The negative correlation between zooplankton biomass and the biomass of golden brown algae is unclear and difficult to explain.

We found a number of more specific relationships between particular trophic groups of plankton (Fig. 7). Different phytoplankton groups were related to each other: cyanobacteria to dinoflagellates, and dinoflagellates to euglenoids. There were other relationships between phytoplankton groups and different trophic groups of zooplankton. Only euglenoids and cryptomonads were correlated with omnivorous ciliates. In general, phytoplankton groups showed more connections with different zooplankton groups and among themselves, but ciliate groups showed more connections among themselves and with zooplankton groups. These simple relationships support the notion that ciliates transfer organic matter to zooplankton. According to the microbial loop concept, the dissolved organic carbon released by phytoplankton is used by bacteria, which are then preyed upon by protozoa that are subsequently consumed by zooplankton [45, 46].

The simple positive correlation observed between the biomass of cyanobacteria and dinoflagellates is supported by laboratory experiments demonstrating allelopathic interactions between dinoflagellates and toxic cyanobacteria [47]. Simple positive relationships between dinoflagellates and euglenoids might be explained as coexistence. We speculate that because both of these organisms are mobile and mixotrophic, they can use alternative methods of feeding and do not compete.

The negative correlation between the biomass of herbivorous zooplankton that feeds on small algae and the biomass of small green algae (<30 μm) are explained by grazing, and the negative correlation between the biomass of herbivorous zooplankton and that of ciliates that feed on bacteria and algae can be explained by competition.

Many studies have suggested that the biomass of some herbivorous zooplankton species (mostly *Daphnia* species) decreases during cyanobacterial blooms [48]. Often a negative correlation between the biomass of herbivorous zooplankton and that of dinoflagellates and cyanobacteria is explained as a lack of a food source for zooplankton. This would seem to make the positive relationships we found between these organisms and cyanobacterial biomass difficult to explain. However, recent reports increasingly suggest that *Daphnia*–cyanobacteria relationships are more complicated than previously thought and that a decrease in the daphnid population during cyanobacterial blooms is not necessarily the result of toxins [49]. Moreover, short-term exposure to toxic cyanobacteria can improve the fitness of *Daphnia magna* for further exposure to toxic prey during development. This trait might be transferred to offspring via maternal effects, or such an adaptation might be clone-specific [50].

The negative correlation between the biomass of herbivorous zooplankton species that feed on big algae (>30 μm) and that of zooplankton species that feed on the seston and bacteria may suggest some unknown type of competition. Animals that feed on the seston and bacteria are an important link in the transfer of carbon from bacterial biomass to macrozooplankton [51, 52], and might compete with ciliates which also transfer organic matter from bacteria in a microbial loop. This possibility will be the focus of our future work.

Predation may also explain the negative relationship between the biomass of predator zooplankton and the biomass of ciliates that feed on bacteria and algae. Copepods, which traditionally have been considered to be herbivores, are in fact omnivores which also feed on heterotrophic protists and are inefficient at feeding on prey less than 5–10 μm in size [53]. Large-bodied copepods can effectively consume protists (heterotrophic nanoflagellates and ciliates), rotifers, and cladocerans [54].

Simple relationships allowed us to outline the trophic network among plankton components in the four small shallow oxbow lakes we studied. The network was underpinned by adding plankton ciliates, which are often neglected in such studies. In general, the relationships indicated the flow of organic matter from phytoplankton to zooplankton and from ciliates to zooplankton.
